# First person – Karen Lange

**DOI:** 10.1242/dmm.048553

**Published:** 2021-01-26

## Abstract

First Person is a series of interviews with the first authors of a selection of papers published in Disease Models & Mechanisms, helping early-career researchers promote themselves alongside their papers. Karen Lange is first author on ‘Interpreting the pathogenicity of Joubert syndrome missense variants in *Caenorhabditis elegans*’, published in DMM. Karen is a postdoc in the lab of Oliver Blacque at University College Dublin, Dublin, Ireland, using genome-editing techniques in worms to investigate ciliopathies, rare genetic disorders caused by defects in cilia.


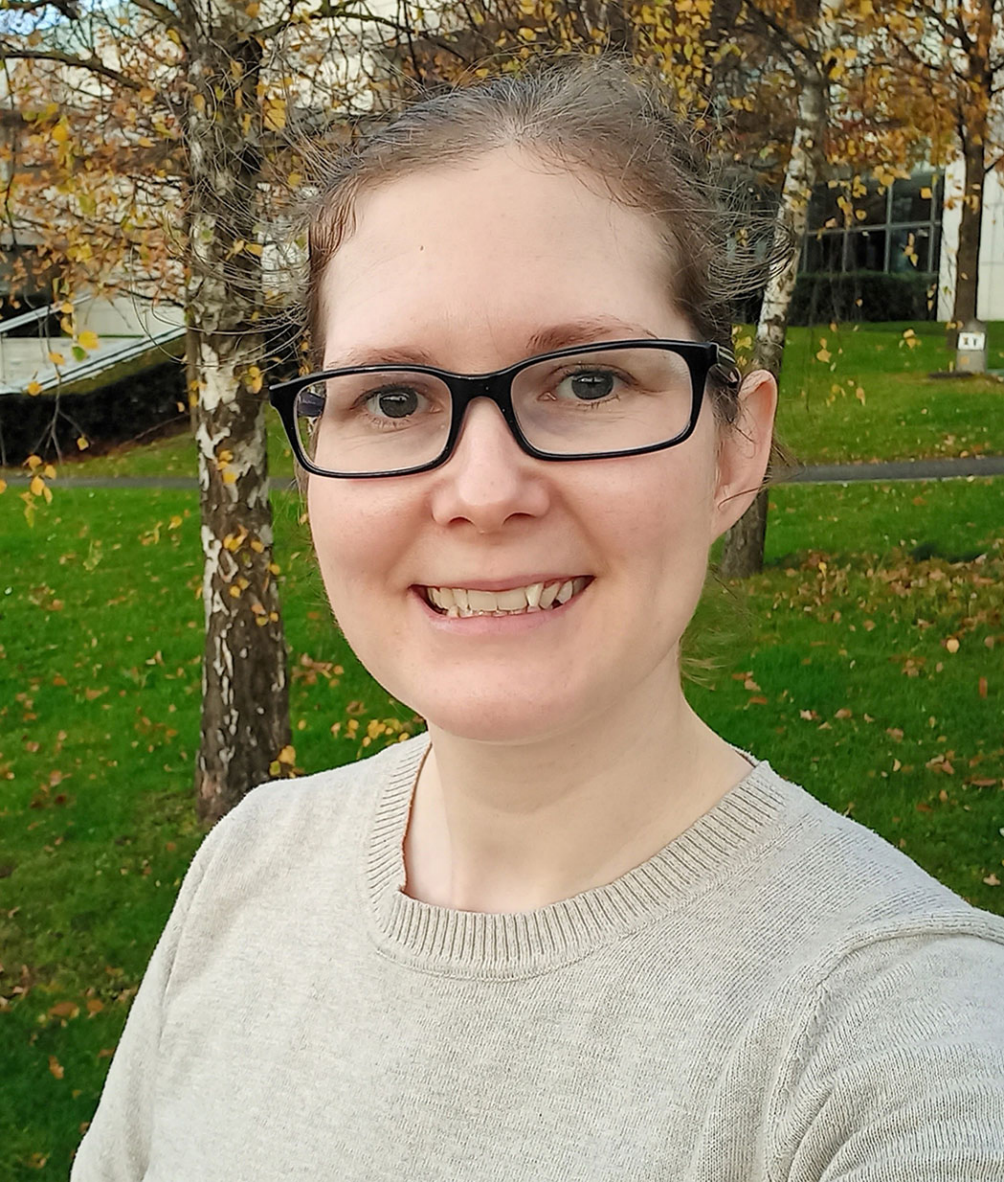


**Karen Lange**

**How would you explain the main findings of your paper to non-scientific family and friends?**

I used CRISPR to genetically modify small worms to have a specific genetic variation that was originally identified in a patient with Joubert syndrome. Joubert syndrome is a rare genetic condition that arises when antenna-like projections on cells do not function properly. These ‘antennae’, called cilia, are found on almost every human cell and are needed to receive signals from the environment. When the cilia do not function properly it affects the entire body. Joubert syndrome has symptoms that affect the brain, eyes, kidneys, liver and skeleton.

The worms that I used in this study do not have eyes, kidneys, liver or a skeleton. However, they do have cilia on some of their cells. When I genetically modified the worms to have the same variation as the patient with Joubert syndrome, I found that the cilia of these worms did not properly sense the environment. These data confirm that the genetic variation does indeed affect cilia. It is important to identify the genetic cause of disorders such as Joubert syndrome because this information can be used for genetic counselling or even to qualify patients for clinical trials.

**What are the potential implications of these results for your field of research?**

This study acts as a proof of principle that CRISPR in *C. elegans* can be used to generate and characterize ciliopathy-associated missense variants. While we only looked at one ciliopathy gene in this study (*mksr-2/B9D2*), I anticipate that these promising results will be broadly applicable to other genes. One clear application of this research would be to model variants of unknown significance in *C. elegans.* This would allow us to assess the pathogenicity of variants and could potentially help clinicians identify pathogenic variants in patients with ciliopathies. Another exciting direction for this research could be to use these engineered worms for drug screens. Some of the experiments we use to assess cilia function, such as the dye-filling assay, can easily be adapted for a large-scale screen. There have been several recent success stories where drug screens in *C. elegans* have identified new treatments for genetic disorders.

“Not all cilia are created equally […]”

**What are the main advantages and drawbacks of the model system you have used as it relates to the disease you are investigating?**

*C. elegans* is a fantastic model organism to study cilia. In worms, cilia are non-essential so we can culture and grow cilia mutants. The short lifespan of *C. elegans* means it is well suited to genetics. I took advantage of that in this research by using genetic crosses to generate compound heterozygous worms that reflect the genotype of the original patient with Joubert syndrome.

*C. elegans* have primary cilia on sensory neurons. In contrast, cilia are found on almost every type of human cell. Not all cilia are created equally; cilia from different tissues and cell types have subtle differences. The primary cilia in worms are excellent to study general cilia biology, but what we learn from worms will likely not directly translate to all cilia.

**What has surprised you the most while conducting your research?**

I am always amazed at how efficiently CRISPR-Cas9 can be used to specifically engineer the genome. This project started because I was curious to see if we could make specific missense mutations in worms. The published protocols seemed straightforward but I was still surprised when we were able to make both mutations on our first try.

“Time is one of the most valuable resources in the lab.”

**Describe what you think is the most significant challenge impacting your research at this time and how will this be addressed over the next 10 years?**

In general, I think that increasing the number of permanent senior scientist positions would greatly benefit science because this would help to retain highly trained scientists in academia. The lack of job security and short-term contracts are a large deterrent from remaining in academic research.

Time is one of the most valuable resources in the lab. Restrictions and lockdowns in response to the COVID-19 pandemic this year meant I had significantly less time in the lab than expected. With greatly reduced lab access this year, progress on projects I am working on has been much slower than planned. As a result, I am concerned that I may not be able to publish these projects before my contract ends next year. In Ireland, government funding has been allocated to provide extensions for researchers and students whose research was interrupted by the pandemic. While there is not enough funding to support everyone, I think this is a great initiative to at least help some of the most affected students and researchers.

**Figure:**
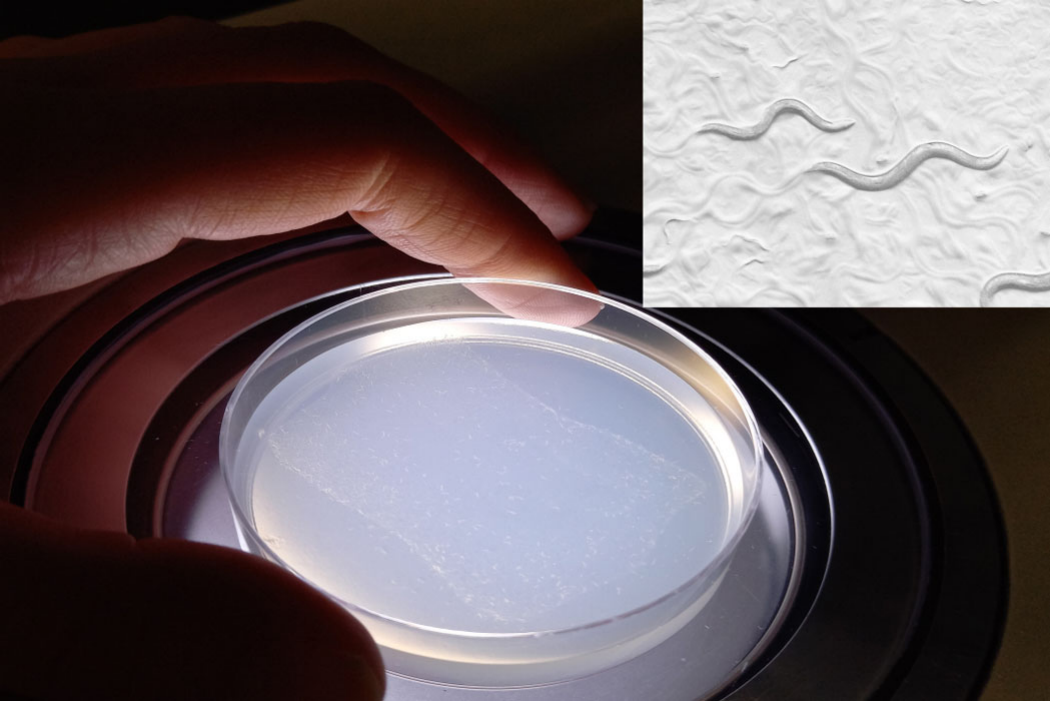
**An agar plate with *C. elegans* growing on it. The inset shows a magnified view of the worms.**

**What changes do you think could improve the professional lives of early-career scientists?**

University College Dublin has a lot of resources available to support postdocs. This includes postdoc-specific career resources such as mock academic interviews, one-on-one career meetings, skills workshops, a wide variety of seminars, and even a course for project management certification. I think all early-career researchers could benefit from having access to resources like these.

**What's next for you?**

My current contract finishes in 2021 and I am considering several possibilities for my next role. I love *C. elegans* and hope I will be able to find a position where I can continue working with worms.
